# Inhibition of soluble epoxide hydrolase in endotoxin induced pig lung injury

**DOI:** 10.3389/fphar.2025.1652349

**Published:** 2025-09-10

**Authors:** Niklas Larsson, Cindy B. McReynolds, Sung Hee Hwang, Debin Wan, Jun Yang, Richard Lindberg, Stefan Lehtipalo, Jonas Claesson, Amanda Irgum Liljeström, Alicia Lind, Anders Brolin, Martin Isaksson Mettävainio, Bruce D. Hammock, Christophe Morisseau, Malin L. Nording

**Affiliations:** ^1^ Anesthesiology and Intensive Care, Department of Diagnostics and Intervention, Umeå University, Umeå, Sweden; ^2^ Department of Entomology and Nematology, and UC Davis Comprehensive Cancer Center, University of California Davis, Davis, CA, United States; ^3^ Department of Chemistry, Umeå University, Umeå, Sweden; ^4^ Department of Medical Biosciences, Umeå University, Umeå, Sweden

**Keywords:** acute respiratory distress syndrome, lung injury, soluble epoxide hydrolase, AEPU, lipid mediators

## Abstract

Pharmacological inhibition of soluble epoxide hydrolase has been shown to attenuate lung injury development in rodents exposed to bacterial lipopolysaccharide. To investigate if these effects can be reproduced in larger animals, we tested soluble epoxide hydrolase (sEH) inhibition using an sEH inhibitor 1-adamantanyl-3-{5-[2-(ethylethoxy)ethoxy]pentyl}urea (AEPU) in a porcine model of lipopolysaccharide-induced acute lung injury. AEPU was selected from 23 sEH inhibitors based on IC50 values and metabolic stability profiles established by a fluorescent based activity assay and porcine liver microsomal test, respectively. Hydrolysis of fatty acid epoxides to their corresponding diols is catalyzed by sEH. Inhibition of sEH reduces this conversion, leading to an accumulation of epoxides relative to diols. Hence, AEPU-treated subjects (n = 9) showed metabolic signs of effective *in vivo* inhibition of the target enzyme reflected in an increased epoxide/diol ratio of 12 (13)-epoxyoctadecenoic acid to 12,13-dihydroxyoctadecenoic acid compared to placebo-treated controls (p = 0.026). However, there was no difference in lung injury development or survival in subjects treated with the rapidly metabolized AEPU compared to placebo-treated controls (n = 10). In conclusion, administration of the soluble epoxide hydrolase inhibitor AEPU did not attenuate endotoxin induced lung injury with lipopolysaccharide in pigs under the severe conditions tested here.

## 1 Introduction

Acute respiratory distress syndrome (ARDS) is a severe inflammatory condition common in intensive care. Despite decades of research, mortality remains high (ARDS Definition Task Force et al., 2012). Well recognized causes of ARDS include primary lung damage and secondary lung injury related to excessive systemic inflammatory response to sepsis, trauma, major hemorrhage and other insults of great diversity (Fanelli et al., 2013). In ARDS development, primary endothelial damage is aggravated by alveolar flooding due to loss of epithelial integrity and accumulation of neutrophils (Matthay and Zemans, 2011). Furthermore, a disturbed balance between factors which promote or inhibit fibrogenesis after the acute lung injury leads to unfavorable remodeling of the lung extracellular matrix. Epithelial apoptosis, mesenchymal cell invasion and protein deposition leave permanent changes in the epithelial lining. Finally, disturbances in properties of epithelial cell proliferation, apoptosis and migration promote fibrogenesis and development of the permanent alveolar damage frequently seen after ARDS (Rocco et al., 2009).

Patients require symptomatic treatment with non-invasive or, more commonly, invasive mechanical respiration (ARDS Definition Task Force et al., 2012). This respiratory support may in itself contribute to additional lung damage through stretch injury to less affected lung regions which are more compliant in the face of positive pressure inflation of the lung. To date, no anti-inflammatory pharmacological treatment of ARDS has shown clear benefit in clinical trials. The disappointing results of clinical trials have not confirmed a number of experimental studies showing benefit of various anti-inflammatory pharmacological agents in animal models (Frank and Thompson, 2010).

Metabolic products of the conversion of arachidonic acid (AA), eicosanoids and other regulatory lipid mediators, are part of the inflammatory cascade (Dennis and Norris, 2015). They display proinflammatory properties (e.g., prostaglandins and thromboxanes) and promote resolution of inflammation (e.g., epoxy fatty acids) (Hammock et al., 2020). In parallel to the AA cascade, other fatty acids, such as eicosapentaenoic acid and docosahexaenoic acid, are metabolized along the same routes, yielding a number of different lipid mediators with important actions in inflammatory signaling (Morisseau and Hammock, 2013). Well-known enzymes responsible for the metabolism of fatty acids are the cyclooxygenases (COX), lipoxygenases (LOX), and cytochrome P450 (CYP). Non-steroidal anti-inflammatory drugs (NSAIDs) target the COX pathway. Lipoxygenase inhibitors have been developed for therapeutic small airway smooth muscle relaxation in asthma. Cytochrome P450 enzymes, forming epoxides, were identified in the 1980s, and have during the last decades received close attention due to their participation in the inflammatory system (Schmelzer et al., 2005; Spector, 2009; Wang and Dubois, 2012).

CYP enzymes metabolize AA into several products, including fatty acid epoxides called epoxyeicosatrienoic acids (EpETrEs). EpETrEs have anti-inflammatory, antifibrotic, and antihypertensive properties (Harris and Hammock, 2013). The biological effects of EpETrEs are in normal physiological circumstances limited in their systemic effects by rapid hydrolyzation by, among other hydrolases, soluble epoxide hydrolase (sEH), which converts the EpETrEs to their corresponding diols, termed dihydroxyeicosatrienoic acids (DiHETrEs) (Spector and Norris, 2007). Pharmacological inhibition of sEH causes accumulation of the EpETrEs, leading to enhanced effects of EpETrEs (Inceoglu et al., 2007). EpETrE regulation could be relevant in the development of several diseases and is studied as a potential new treatment in many conditions where inflammation is a contributing or crucial element, including in chronic obstructive pulmonary (COPD) and asthma, but also for diabetes, chronic pain, and ischemic heart disease (Morisseau and Hammock, 2013; Wang and Dubois, 2012; Wang et al., 2021; Turnbull and Chapman, 2024; Yu et al., 2023; Yang et al., 2015; Luo et al., 2021).

Furthermore, CYP enzymes generate fatty acid epoxides called epoxyoctadecenoic acids (EpOMEs) from linoleic acid (LA) (Hildreth et al., 2020). These epoxides were originally known as leukotoxins, but it was later discovered that their downstream diols were in fact responsible for the toxic effects (Sugiyama et al., 1987; Zheng et al., 2001; Moghaddam et al., 1997). The epoxide 9,10-EpOME was initially shown to increase in ARDS in association with burns (Ozawa et al., 1991; Kosaka et al., 1994). However, experimentally EpOMEs appear to have little direct effect on lung injury, whereas their conversion by sEH to their corresponding diols produces the highly active diols called dihydroxyoctadecenoic acid (DiHOMEs) that have been clearly implicated in the pathogenesis of experimental lung injury (Zheng et al., 2001; Kosaka et al., 1994), as well as in COVID-19 (McReynolds et al., 2021). In regard to burn injury, recent data revealed DiHOMEs as a driver for the pathological inflammation in burn injury, rather than the EpOMEs (Bergmann et al., 2022).

Taken together, the increase of endogenous epoxy-fatty acid levels and blocking of fatty acid diol formation by sEH inhibition serve as a basis for the design of new clinical interventions (Moghaddam et al., 1997). In a murine model of lipopolysaccharide (LPS) induced acute lung injury, sEH inhibition led to significant positive effects, including attenuated morphological lung injury and improved survival (Zhou et al., 2017). LPS is an important part of the outer surface membrane in a majority of Gram-negative bacteria. It is a potent activator of the innate immune system leading to recruitment and activation of myeloid cells, in the end triggering a number of systemic responses leading to septic shock. Endotoxin challenge is frequently used as a model for sepsis and to induce ARDS in animal experiments (Lopez-Aguilar et al., 2010; Wyns et al., 2015). However, sEH inhibition in models of ARDS has not been studied in larger animals.

Thus, we hypothesized that sEH inhibition would attenuate acute diffuse inflammatory lung injury development assessed by oxygenation and histological scoring in a non-rodent large animal experimental lung injury model. We aimed to test this using 1-adamantanyl-3-{5-[2-(ethylethoxy)ethoxy]pentyl}urea (AEPU) (Kim et al., 2007) in a porcine model with endotoxin-induced lung injury, and compare the outcomes to animals with the same lung injury treated with placebo.

## 2 Materials and methods

### 2.1 Animals

22 Yorkshire-Swedish landrace pigs were used in accordance with the EU Directive 2010/63/EU for animal experiments and with ethical approval from the Animal Ethical use in Research Review Authority in Umeå, Sweden (31-12910/12). The animals were bred for purpose at the Forslunda Agricultural School in Umeå, Sweden. The pigs were pairwise kept in 8 m^2^ pens before start of experiments and were fed twice daily with a commercially available pig diet without antimicrobials, and with free access to water. Photoperiods were 12:12 h light:dark. Room temperature in their stall was 20 °C ± 1 °C.

### 2.2 Animal preparation

After overnight fasting, but with free access to water and hay, animals were pre-medicated with ketamine (Ketaminol vet^®^ 100 mg/mL; Intervet International B.V, Boxmeer, Netherlands), xylazine (Rompun^®^ vet 20 mg/mL; Bayer Animal Health, Copenhagen, Denmark) and atropine (Atropin Mylan 0.5 mg/mL; Mylan AB, Stockholm, Sweden) by intramuscular injection before an auricular vein was cannulated. Anesthesia was induced with ketamine and midazolam (Midazolam Hameln 5 mg/mL, Hameln Pharmaceuticals, Hameln, Germany) and maintained with ketamine, midazolam, xylazine and fentanyl (Fentanyl 0.05 mg/mL, B. Braun Medical AB, Danderyd, Sweden). The animals were orotracheally intubated and ventilated with tidal volume 8–9 mL/kg, PEEP 8 cmH_2_O, and a respiratory rate set to achieve an end-tidal carbon dioxide of 5–5.5 kPa.

**FIGURE 1:**
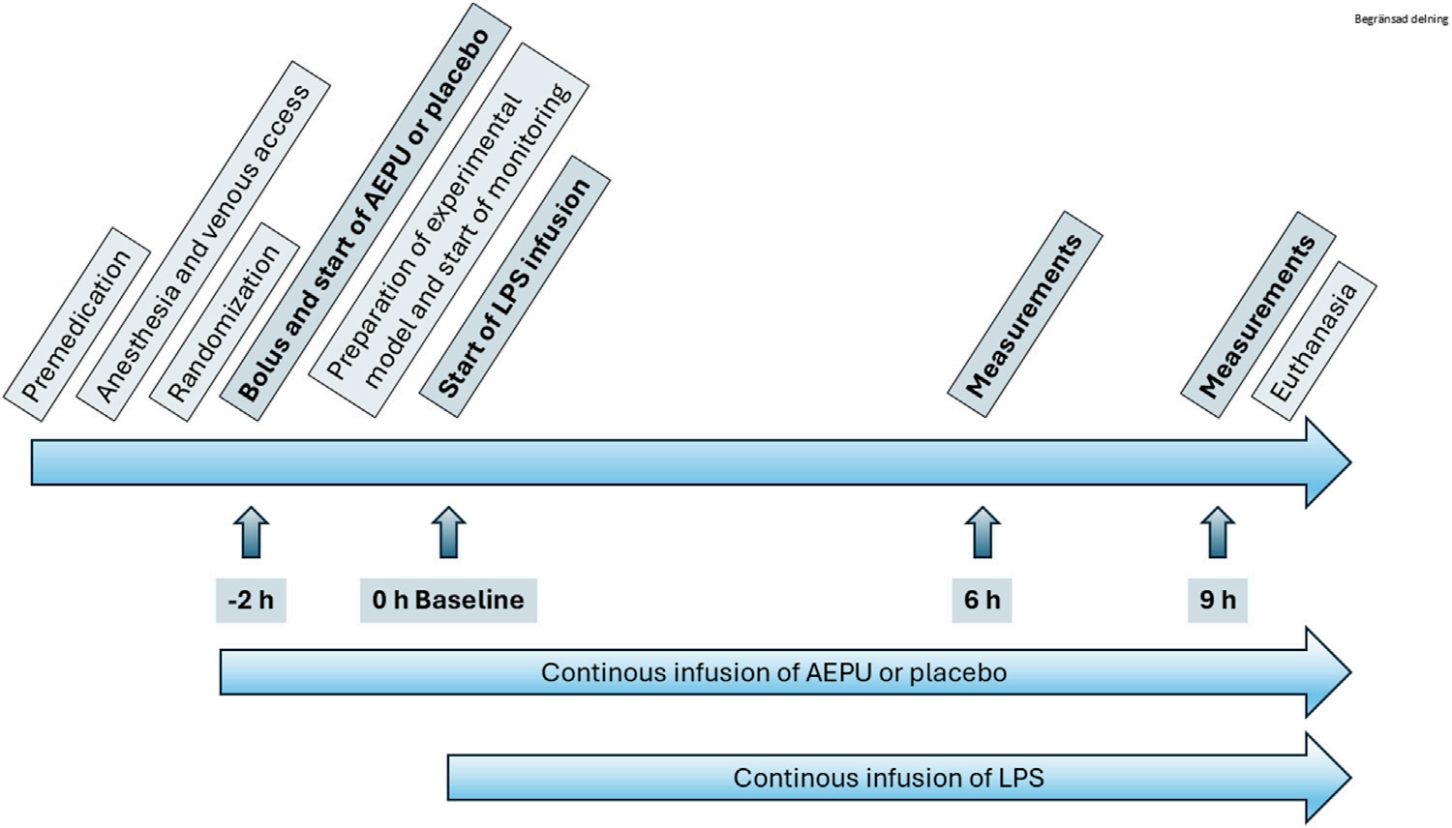
Study overview. Flow chart of study protocol including indication of start of 1-adamantanyl-3-{5-[2-(ethylethoxy)ethoxy]pentyl}urea (AEPU) treatment and lipopolysaccharide (LPS) infusion.

One animal served as a pharmacokinetic pilot, with arterial and venous catheters placed in a cut-down in the neck. The remaining 21 animals were used for the randomized study (Figure 1). After intubation and start of mechanical ventilation, a second auricular vein cannula was placed and infusion of either AEPU or placebo according to randomization was started. After 2 h of infusion of AEPU or placebo, an infusion of LPS was started. A bolus of 0.1 mg/kg was administered during 30 min after which the infusion rate was adjusted to 0.01 mg/kg/h. During the time between start of AEPU or placebo and LPS infusion, neck dissection was performed, exposing the v jugularis externa and carotid artery. A pulmonary artery introducer and an arterial cannula were placed in vein and artery respectively, and a pulmonary artery catheter introduced through the introducer and the tip placed in the correct position in the pulmonary artery placement guided by continuous invasive pressure monitoring or, when difficult, assisted by fluoroscopy. All surgical procedures were performed under sterile conditions.

After completion of the study protocol, the animals were euthanized, still under general anesthesia, by injection of an overdose of Tiopental (Pentocur^®^ 0.5 g/vial, Abcur AB, Helsingborg, Sweden) followed by 40 mmol of potassium chloride. Asystole was confirmed with ECG.

### 2.3 Randomization

Each day of the study, two animals were prepared and randomized to receive either the AEPU or the placebo infusion. One designated person prepared the solutions, randomly assigning them the letter A or B. The solutions were then, in turn, randomly assigned to one animal each by another person unaware of the content of solution A or B. The person preparing the solutions did not actively participate in the treatment part of the experiment.

### 2.4 Inhibitor selection

The chemical structures of the 23 compounds tested in this study are given in Table 1. All of the compounds were a subset of the Hammock laboratory small molecule library of sEH inhibitors (Kim et al., 2004; Jones et al., 2006; Hwang et al., 2007; Rose et al., 2010; Tsai et al., 2010), except one that was used in a clinical study for COPD treatment (Wang et al., 2012). They all contained urea as the central pharmacophore, which binds to the active site of sEH. The subset of compounds was selected based on adequate pharmacokinetic data in other species (Rose et al., 2010; Tsai et al., 2010; Guedes et al., 2018; Ulu et al., 2012), and IC_50_ < 20 nM on the affinity purified human recombinant sEH (Sun et al., 2021).

**TABLE 1:** Soluble epoxide hydrolase (sEH) inhibitors. Chemical structures, inhibition potency (IC_50_ values), and liver microsomal stability of soluble epoxide hydrolase inhibitors in pig. 1-adamantanyl-3-{5-[2-(ethylethoxy)ethoxy]pentyl}urea (AEPU) corresponds to compound number 950. Compound number refers to internal reference in the Hammock Laboratory’s small molecule library of sEH inhibitors.

Entry	Compound number	Chemical structure	Pig sEH IC_50_ (nM)	Liver microsomal stability: Amount remaining after 35 min (% of initial)
1	2634	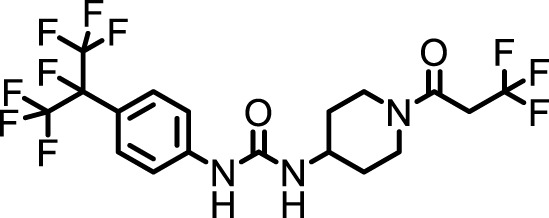	2	*n.a*
2	2633	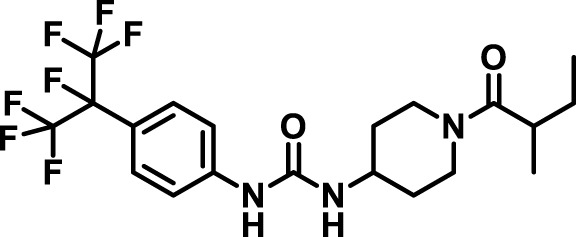	4	58 ± 3
3	700	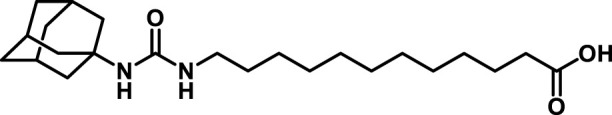	4	1 ± 0.1
4	1671	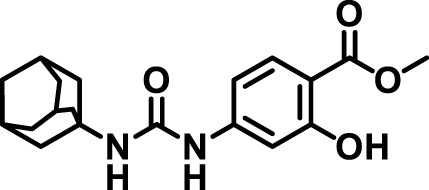	5	*n.a*
5	950	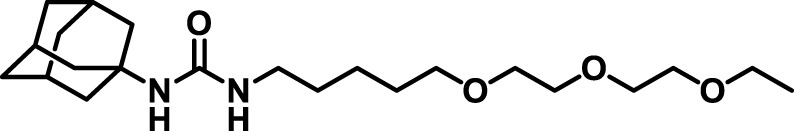	5	1 ± 0.5
6	1153	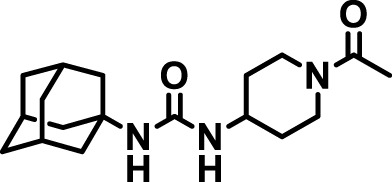	15	*n.a*
7	1686	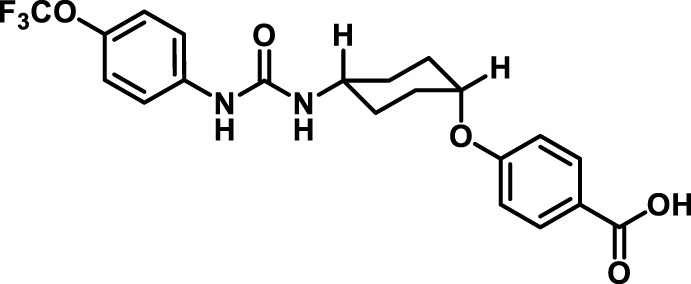	19	*n.a*
8	2385	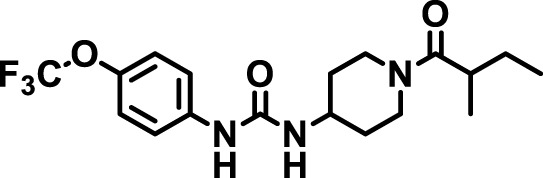	19	55 ± 3
9	2391	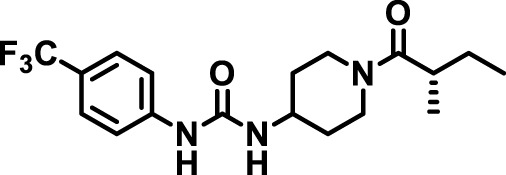	20	*n.a*
10	2383	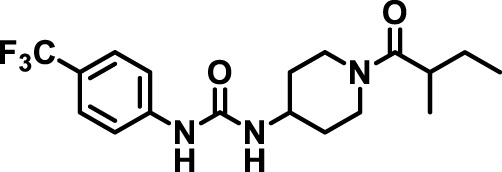	26	*n.a*
11	1728	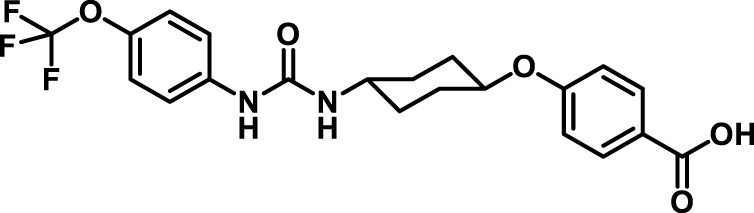	34	78 ± 3
12	2422	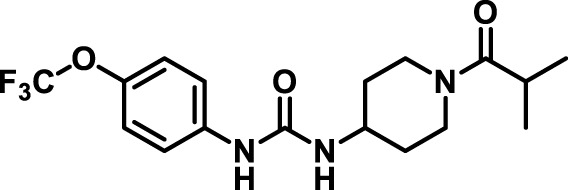	38	*n.a*
13	1663	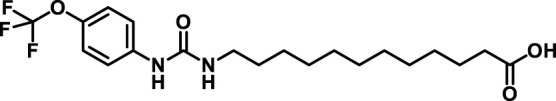	40	*n.a*
14	2714	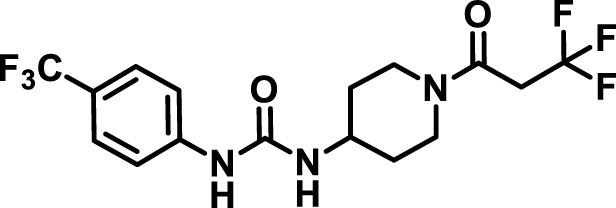	44	*n.a*
15	2946	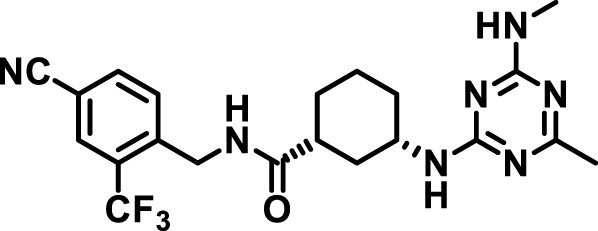	48	66 ± 1
16	1471	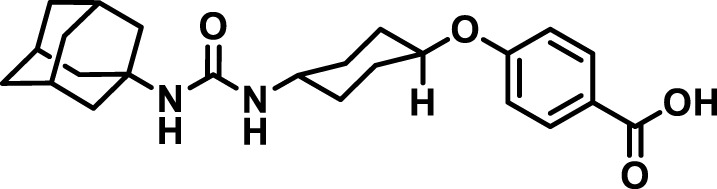	60	*n.a*
17	2413	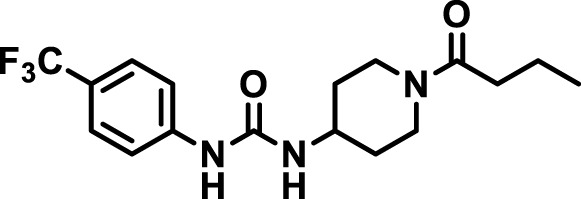	60	*n.a*
18	2389	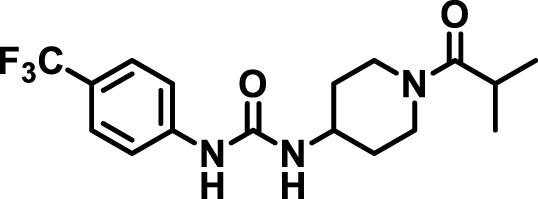	70	*n.a*
19	1770	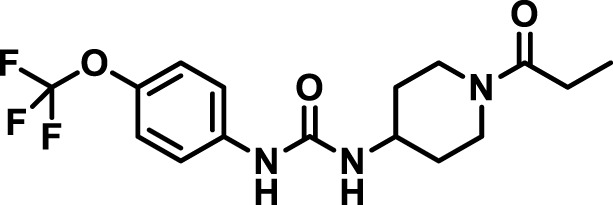	100	85 ± 4
20	59	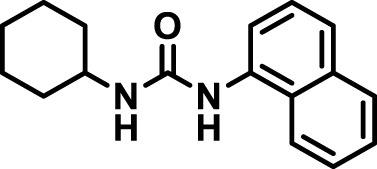	105	*n.a*
21	2421	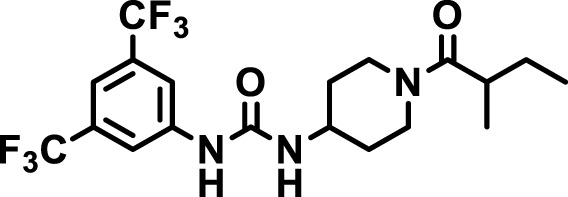	107	*n.a*
22	1555	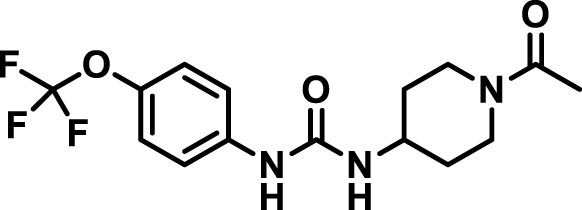	197	*n.a*
23	1709	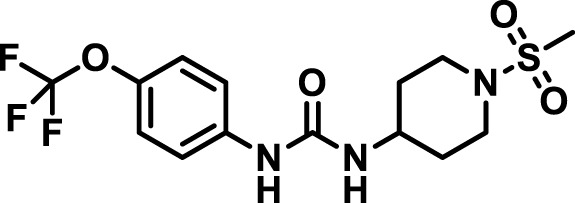	1,291	*n.a*

n.a. = not analyzed.

### 2.5 Determination of inhibitor potencies

Recombinant porcine sEH was expressed and purified as described previously (Newman et al., 2004). A fluorescent assay using MNPC (cyano (6-methoxy-naphthalen-2-yl)methyl trans-[(3-phenyloxiran-2- yl)methyl] carbonate) as substrate was performed to determine inhibition potencies expressed as IC_50_ values (Morisseau and Hammock, 2007). The IC_50_ value was defined as the inhibitor concentration which reduces the enzymatic activity of pig sEH by 50%. In brief, pig sEH was incubated for 5 min at 37 °C with each one of 23 inhibitors in triplicate for each concentration tested. Then, substrate ([S]_final_ = 5 µM) was added. The amount of the fluorescent product (6-methoxynaphthaldehyde) was measured in a microplate reader M2 (Molecular Devices., CA, United States of America) at 37 °C in kinetic mode for 10 min with measurement every 30 s (λ_ex_ = 330 nm, λ_em_ = 465 nm, λ_cutoff_ = 430 nm). Inhibition potency was calculated by plotting log inhibitor concentration versus % inhibition (100 × [diol_w/ inhibitor_]/[diol_w/o inhibitor_]). Inhibitor concentrations in the range of 1–50,000 nM were tested, including at least two points above and two points below the IC_50_. Inhibitor quantification was performed as previously described (Ulu et al., 2012).

### 2.6 Liver microsomal assay

To further characterize the inhibitors with regard to suitability for administration in pigs, a microsomal assay was used to determine metabolic stability (Ackley et al., 2004) for a subset of eight inhibitors chosen for their potency and availability. Microsomal preparations from pig liver were purchased from XenoTech (Kansas City, KS, US). Microsomes, buffer and NADPH generating system were combined, and then the inhibitor was added. The reaction was stopped after 35 min with ice cold acetonitrile containing internal standard (12-[[(cyclohexylamino)carbonyl]amino]-dodecanoic acid). The amount left of parent inhibitor was determined with LC-MS/MS.

### 2.7 Preparation of AEPU and placebo solutions

AEPU was synthesized according to Kim et al. (Kim et al., 2007), delivered in 60 mg vials and dissolved in 30 mL pure ethanol as a co-solvent, of which either 20 mL was added to 980 mL of sodium chloride 9 mg/mL or 8 mL–392 mL sodium chloride, creating a solution of 0.04 mg/mL AEPU and 2% ethanol. The placebo infusion was prepared by adding either 20 mL of pure ethanol to 980 mL of sodium chloride 9 mg/mL or 8 mL of pure ethanol to 392 mL sodium chloride, creating a 2% ethanol solution. Each animal received an infusion of either 0.1 mg/kg/h AEPU or the corresponding volume of placebo solution.

### 2.8 Lipopolysaccharide solution

A total of 200 mg of prefabricated lipopolysaccharides (LPS, *Escherichia coli* serotype 0,111:B4, Sigma-Aldrich (Product no L4130), St. Louis, MO, United States of America) was dissolved in 40 mL of sterile water, carefully mixed and filtered, creating a solution of 5 mg/mL. This preparation was stored frozen (−20 °C) in 1 mL partitions. For each animal, 2 mL of the thawed solution was mixed and diluted in 18 mL of sodium chloride, finally creating a 0.5 mg/mL LPS solution for infusion.

### 2.9 Collection of observed variables

All animals were monitored with pulse oximetry, heart rate, ECG, systemic arterial blood pressure (ABP), pulmonary arterial pressure (PAP), central venous pressure (CVP) temperature, tidal volumes, respiratory rate and end tidal carbon dioxide. At baseline, and at 3 h, 6 h and at the end of the experiment, pulmonary capillary wedge pressure (PCWP) was measured together with arterial and venous blood gas analyses, as well as cardiac output calculations. Routine chemistry, including creatinine, bilirubin, glucose, electrolytes were collected at baseline and at the end of the experiment (Vetscan VS2^®^, Abaxis, Griesheim, Germany). Blood samples for measurement of AEPU plasma concentrations, as well as oxylipins (*i.e.,* EpETrEs and their DiHETrE corresponding diols as well as related compounds with other polyunsaturated fatty acid precursors), were collected at baseline, 6 h and at the end of the experiment. Oxylipin analysis was performed according to a previously published protocol (Gouveia-Figueira and Nording, 2015).

### 2.10 Lung biopsies

After euthanasia, the chest was opened and two approximately 2 × 1 × 1 cm pieces of lung tissue was cut from the left lung 3 cm from the hilus. One piece was immediately put in formaldehyde solution for later routine preparation for histological evaluation in hematoxylin and eosin staining. A pathologist, blinded for treatment group, evaluated the severity of lung injury in specimens on a four graded scale: 0 = no lung injury, 1 = mild lung injury, 2 = moderate lung injury and 3 = severe lung injury.

The second piece of lung tissue was immediately frozen for later assessment of wet to dry (W/D) ratio. The frozen sections were weighed and then put in a 70 °C dryer (Mushroom and Fruit Dehydrator, Clas Ohlson, Insjön, Sweden) for 72 h. The W/D ratio was calculated after recording the dry weight.

### 2.11 Statistical analysis

For the sample size justification, we anticipated a P/F ratio in the control group of 25 kPa (±10) and a clinically relevant increase to 40 kPa in the treatment group. This meant nine subjects in each group to achieve 90% power of detecting a difference of this size given α = 0.05. Anticipating that not all experiments would reach completion, we used a sample size of 22. The results were analyzed using SPSS Statistics, version 24 (IBM Corp., Armonk, N.Y., United States). Normality was examined with the Shapiro-Wilk test, together with examination of histograms of data. Continuous variables were compared using Student’s t-test or Mann Whitney U test, as appropriate. Categorical variables were compared using Fischer’s exact test. Statistical significance was set at α < 0.05. Oxylipins with at least 80% of observations above limit of detection at both timepoints were included in pairwise comparisons of ratios and fold-change evaluations where observations below limit of detection (LOD) were substituted with 0.5 x LOD.

## 3 Results

### 3.1 Inhibition potency and metabolic stability of sEH inhibitors

The suitability for administration of AEPU in pig was confirmed by a fluorescent activity assay for determination of IC_50_ (5 nM). An additional 22 sEH inhibitors were tested to investigate their suitability as candidates in porcine model. The results showed IC_50_-values in the range of 2–1291 nM. Globally, the potency results agree with results found with sEH from other large animals (cat, dog, and horse) (Shihadih et al., 2020). The metabolic stability, assessed by a microsomal assay to determine clearance rate of the drug, range between 0.5%–85% left after 35 min (Table 1). For inhibitors with low metabolic stability, such as AEPU, continuous infusion is required. AEPU displayed a number of favorable properties, including low melting point and high-water solubility, compared to other sEH inhibitors in earlier large animal work (Ulu et al., 2012), but due to the polyethoxy side chain and adamantly group it is rapidly metabolized.

### 3.2 Pharmacokinetic pilot investigation in one animal

**FIGURE 2:**
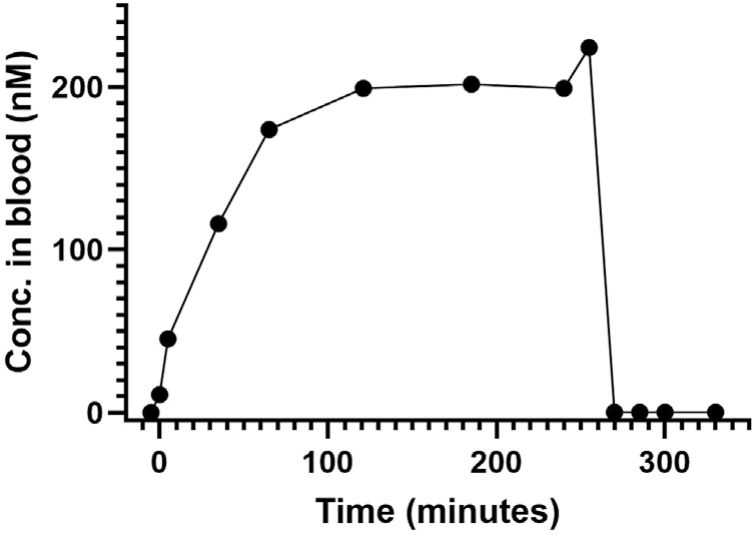
AEPU in pilot animal. Blood concentration (nM) of 1-adamantanyl-3-{5-[2-(ethylethoxy)ethoxy]pentyl}urea (AEPU) in pilot animal using continuous infusion.

Infusion of AEPU at 0.1 mg/kg/h produced a progressive rise in blood concentration reaching steady state of approximately 200 nM at 120 min, maintained with only minor variations until the termination of infusion at 255 min. The elimination of AEPU after termination of infusion was rapid, showing complete elimination within 15 min (Figure 2).

### 3.3 Baseline characteristics in main study

One animal unexpectedly proved impossible to intubate and died due to hypoxia prior to randomization. One animal suffered a bilateral tension pneumothorax and died after randomization in circulatory arrest prior to the start of placebo infusion. The remaining 19 animals were included in the primary analysis. There were no statistically significant differences between the intervention group and the control group at baseline (Table 2).

**TABLE 2:** Study group characteristics. Baseline characteristics of study groups; AEPU treated and placebo controlled. Continuous variables were compared with independent samples t-test or Mann Whitney U test, as appropriate. Nominal variables were compared using Fischer’s exact test.

Variable	AEPU	Control	p
Weight (kg)	30 (29–31)	29 (28–37)	0.60
% Male	70	78	0.63
Hemoglobin (g/L)	91 (90–96)	94 (93–95)	0.74
Creatinine (μmol/L)	71 (62–71)	71 (71–80)	0.32
Bilirubin (μmol/L)	5.1 (3.4–5.1)	5.1 (5.1–5.1)	0.50
Temperature (°C)	38.6 (38.1–39.1)	38.9 (38.6–39.7)	0.13
pH	7.46 (7.44–7.48)	7.44 (7.41–7.44)	0.16
PaCO_2_ (kPa)	4.9 (4.7–5.1)	4.6 (4.5–5.0)	0.80
P/F (kPa)	72 (68–73)	69 (65–71)	0.16
MPAP (mmHg)	25 (24–28)	25 (20–26)	0.25
MAP (mmHg)	91 (74–108)	83 (81–84)	0.66
PCWP (mmHg)	17 (15–18)	15 (14–17)	0.24
SVR (dynes*sec/cm^5^)	1506 (1154–1718)	1624 (1045–1742)	0.94
PVR (dynes*sec/cm^5^)	171 (157–207)	165 (163–267)	1.00
CVP (mmHg)	15 (14–17)	16 (15–18)	0.73
PIP (cmH_2_O)	25 (24–25)	25 (24–26)	0.45
Compliance (ml/cmH_2_O)	11.2 (10.9–11.5)	10.8 (10.4–13.3)	0.36
SvO_2_ (%)	71 (66–74)	72 (62–72)	0.55

Abbreviations: AEPU, 1-adamantanyl-3-{5-[2-(ethylethoxy)ethoxy]pentyl}urea; PaCO_2_, partial pressure of carbon dioxide in arterial blood; P/F, ratio of arterial partial pressure of oxygen to fraction of inspired oxygen; MPAP, mean pulmonary artery pressure; MAP, mean arterial pressure; PCWP, pulmonary capillary wedge pressure; SVR, systemic vascular resistance; CVP, central venous pressure; PIP, peak inspiratory pressure; SvO_2_, saturation of oxygen in venous blood.

### 3.4 AEPU concentrations in LPS-treated pigs

**FIGURE 3:**
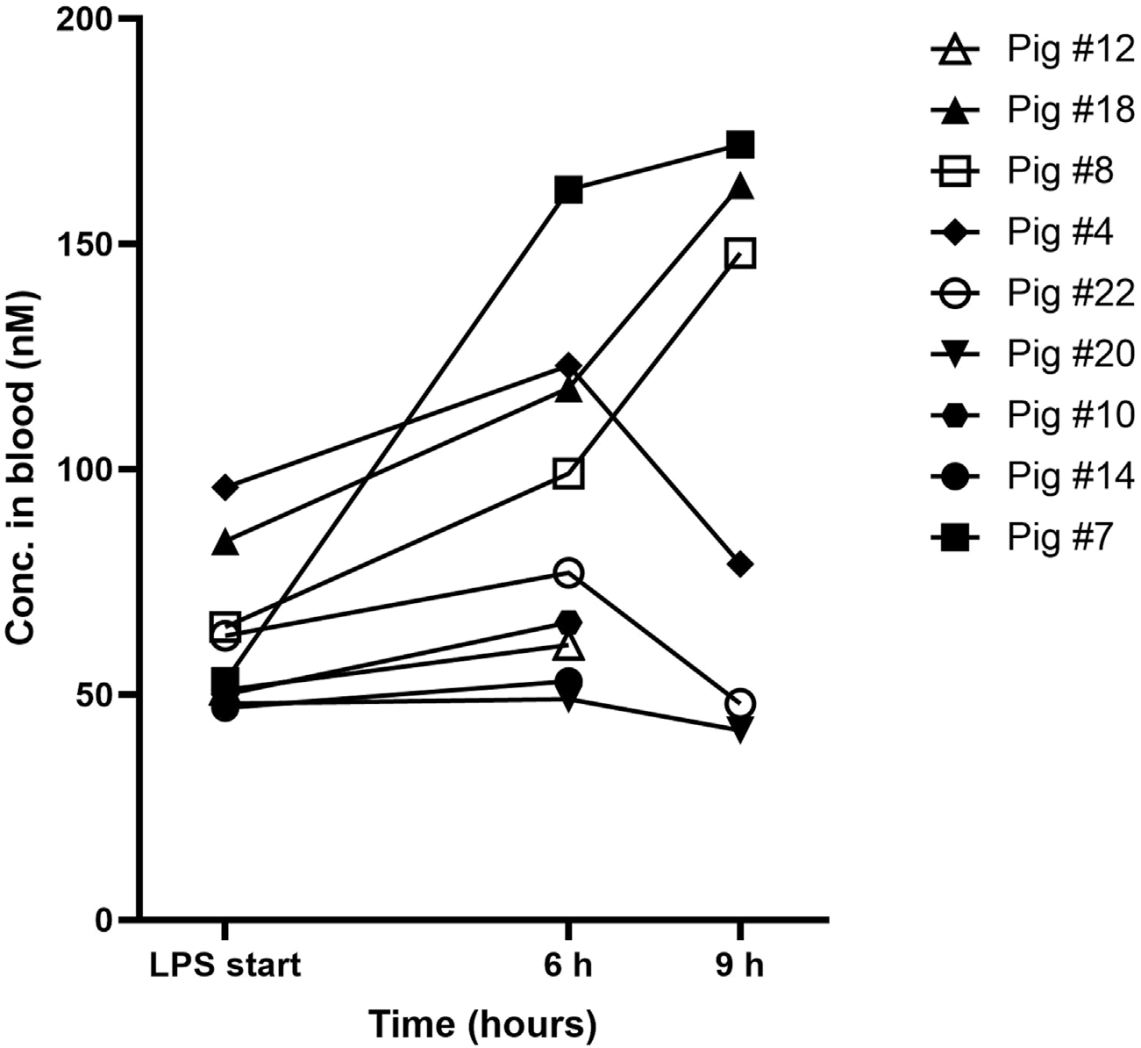
AEPU in LPS-treated pigs. Blood concentrations (nM) of 1-adamantanyl-3-{5-[2-(ethylethoxy)ethoxy]pentyl}urea (AEPU) in lipopolysaccharide (LPS)-treated animals using continuous infusion.

Blood concentrations of AEPU are shown at baseline, 6 h and 9 h in Figure 3. As expected, none of the control animals showed detectable levels of AEPU in blood at any time. All of the actively treated animals had detectable blood concentrations of AEPU throughout the experiments of at least 10 times the IC_50_-value, except for one observation (pig #20 at 9 h).

### 3.5 Results after treatment with AEPU or placebo

All measurements except serum creatinine and serum bilirubin were measured at baseline and after 3 h, 6 h and 9 h treatment with active substance or placebo. Creatinine and bilirubin were measured at baseline and at the end of experiment. Some animals (40% of pigs receiving AEPU and 33% of controls) did not survive the entire experimental protocol. Comparisons between groups have been made using the last available measurements for each individual animal (Table 3). There were no significant differences in outcome between the two groups.

**TABLE 3:** Characteristics at end of experiments**.** Final observations of study groups after AEPU treatment compared to the control group receiving placebo. Continuous variables were compared with independent samples t-test or Mann Whitney U test, as appropriate. Ordinal variables were compared with Mann Whitney U test. Nominal variables were compared using Fischer’s exact test.

Variable	AEPU	Control	p
Premature death (%)	40	33	0.57
Fluid administration total (mL)	5785 (5080–7210)	5090 (4180–6080)	0.44
Urine output (mL)	700 (320–1200)	825 (575–925)	0.89
Hemoglobin (g/L)	116 (107–128)	102 (99–118)	0.13
Creatinine (μmol/L)	133 (102–152)	133 (115–159)	0.48
Bilirubin (μmol/L)	11.1 (8.6–18.8)	8.6 (6.8–22.2)	0.66
Temperature (°C)	38.5 (38.0–40.1)	38.9 (38.9–40.1)	0.36
pH	7.16 (7.07–7.26)	7.25 (7.14–7.29)	0.32
PaCO_2_ (kPa)	5.8 (4.9–7.1)	5.8 (4.5–6.0)	0.45
P/F (kPa)	22 (11–42)	31 (28–47)	0.40
MPAP (mmHg)	46 (35–50)	42 (35–42)	0.46
MAP (mmHg)	66 (55–97)	78 (55–93)	0.91
PCWP (mmHg)	18 (16–21)	18 (16–20)	0.91
SVR (dynes*sec/cm^5^)	1314 (1089–2375)	1230 (958–1457)	0.48
PVR (dynes*sec/cm^5^)	643 (390–1057)	486 (345–827)	0.36
CVP (mmHg)	16 (15–17)	20 (16–24)	0.37
PIP (cmH_2_0)	33 (29–37)	33 (29–37)	0.85
Compliance (ml/cmH_2_O)	7.9 (7.3–9.7)	7.9 (7.0–10.7)	0.78
SvO_2_ (%)	55 (25–66)	55 (48–59)	0.59
W/D ratio	6.9 (6.0–9.0)	6.1 (5.3–6.5)	0.24
Histological injury score	2.7 (2–3)	2.6 (2–3)	0.65

Abbreviations: AEPU, 1-adamantanyl-3-{5-[2-(ethylethoxy)ethoxy]pentyl}urea; PaCO_2_, partial pressure of carbon dioxide in arterial blood; P/F, ratio of arterial partial pressure of oxygen to fraction of inspired oxygen; MPAP, mean pulmonary artery pressure; MAP, mean arterial pressure; PCWP, pulmonary capillary wedge pressure; SVR, systemic vascular resistance; PVR, pulmonary vascular resistance; CVP, central venous pressure; PIP, peak inspiratory pressure; W/D, wet/dry; SvO_2_, saturation of oxygen in venous blood.

### 3.6 Repeated oxylipin measurements

**FIGURE 4:**
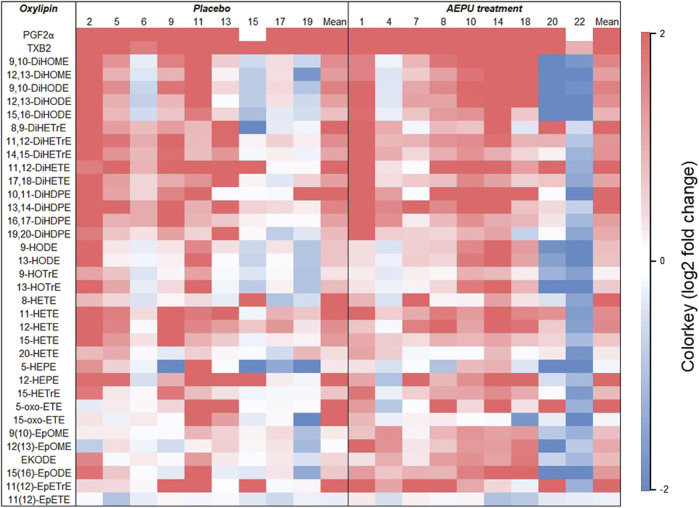
Change in oxylipin concentrations. Heatmap representing the log2 fold-change values of oxylipin levels between baseline and 6 hours in placebo animals (#2, 5, 6, 9, 11, 13, 15, 17, 19) and animals treated with 1-adamantanyl-3-{5-[2-(ethylethoxy)ethoxy]pentyl}urea (AEPU; #1, 4, 7, 8, 10, 14, 18, 20, 22). The value of fold-change between timepoints for each animal is indicated by the colored scale with red representing a value > 2 (a four-fold increase) and blue representing a value < 2 (a four-fold decrease).

Oxylipin levels increased over the course of the experiment in most animals, independent of treatment with AEPU or placebo (Figure 4). Oxylipins with detectable levels in at least 80% of the samples in one of the groups, and at both timepoints, were included in the analysis.

For assessment of target engagement, three pairs of epoxide/diol ratios met the criteria for inclusion in analysis of the results (more than 80% of samples had detectable levels in at least one of the groups). Of these, the ratio of 12(13)-EpOME/12,13-DiHOME was increased at 6 h in the group that received AEPU (Table 4).

**TABLE 4:** sEH substrates and products. Oxylipin levels (nM) and epoxide/diol ratios at 6 hours expressed as mean (standard deviation) in placebo animals and animals treated with 1-adamantanyl-3-{5-[2-(ethylethoxy)ethoxy]pentyl}urea (AEPU). Groups were compared with independent samples t-test.

Oxylipin	Placebo Mean (SD)	AEPU Mean (SD)	Hedges’ G (95% CI)	p
15(16)-EpODE (nM)	14.36 (11.84)	26.10 (27.79)	0.49 (−0.40–1.36)	0.282
15,16-DiHODE (nM)	32.43 (21.79)	38.08 (23.02)	0.23 (−0.64–1.09)	0.610
Ratio 15(16)-EpODE/15,16-DiHODE	0.44 (0.30)	0.57 (0.38)	0.33 (−0.55–1.19)	0.468
9(10)-EpOME (nM)	0.67 (0.27)	1.47 (1.12)	0.86 (−0.06–1.76)	0.065
9,10-DiHOME (nM)	4.48 (3.43)	5.21 (3.00)	0.20 (−0.66–1.06	0.648
Ratio 9(10)-EpOME/9,10-DiHOME	0.28 (0.20)	0.43 (0.41)	0.44 (−0.44–1.30)	0.334
12(13)-EpOME (nM)	1.09 (0.42)	5.12 (4.71)	1.06 (0.12–1.98)	0.027
12,13-DiHOME (nM)	17.80 (14.30)	17.24 (10.55)	−0.41 (−0.90 - 0-82)	0.927
Ratio 12(13)-EpOME/12,13-DiHOME	0.13 (0.10)	0.40 (0.30)	1.07 (0.13–1.99)	0.026

## 4 Discussion

Although previous work has shown attenuation of lung injury development resulting from sEH inhibition during LPS treatment in smaller animals, the present study did not show this effect. There was no improvement in oxygenation, survival or histological lung injury, although *in vivo* inhibition of sEH was implied by an increased epoxide/diol ratio of 12 (13)-EpOME to 12,13-DiHOME in the AEPU-treated group. The selected sEH inhibitor, AEPU, based on favorable properties among the 22 tested for IC_50_ and metabolic stability in pig, was demonstrated to effectively inhibit pig sEH *in vitro*. The dose of AEPU to completely inhibit sEH in pigs was not previously known, although earlier animal experiments suggest that blood concentrations of inhibitor more than five times IC_50_ are enough to effectively inhibit the enzyme *in vivo* effect. In this study, the main study animals all had blood concentrations of AEPU lower than the very stable blood concentrations of AEPU in the pilot animal (once steady state was reached under continuous infusion of the drug) using the same dosing by weight, however, they all had AEPU blood concentrations above or very close to 10 times the IC_50_. This implies that the lack of effect of the drug on LPS-induced lung injury was not from under-dosing (Imig et al., 2005). In most animals, AEPU blood concentrations increased during the experiment. One possible explanation for this may be acutely failing organ function in the setting of severe systemic inflammation with multiple organ dysfunction during the course of the experiments.

The reason for the lack of efficacy is unclear. While the severity of the inflammation caused by LPS here may be too high for a sEH inhibitor alone to be effective, this level of exposure is a clinically relevant model for acute inflammation. Several studies suggest that sEH inhibition could be more effective at reducing and regulating chronic inflammation, especially in the lung (Felipe et al., 2023; Zhang et al., 2023a).

It is recognized that, in the context of studying sepsis, a 9-h observation period is by no means long. Despite this, earlier rodent studies have demonstrated benefits of sEH inhibition within this timeframe (Zhou et al., 2017; Zhang et al., 2023b). Given that the animals were pretreated with AEPU before LPS infusion, it is reasonable to expect that any beneficial effect of the drug would be detectable within the study period.

Regarding the effect of LPS treatment on individual oxylipins, a shift in levels was observed in both the AEPU and placebo groups, as illustrated in the heatmap. It highlights a pattern of elevated levels of especially PGF_2α_ and TXB_2_ – both known proinflammatory lipid mediators–along with several other oxylipins. This pattern supports the notion that biosynthesis and systemic release of free fatty acids from cell membranes occurred over the course of the experiment, from the initiation of LPS infusion to 6 h later, with AEPU having only a minor effect on this process suggesting that the LPS treatment overwhelmed the system to a similar extent in both groups, and that AEPU failed to effectively counteract this proinflammatory response at the tested dose. It should be noted that interpretations of oxylipin levels are hampered by significant interindividual variations.

However, the increased 12 (13)-EpOME/12,13-DiHOME ratio after 6 hs in the AEPU group compared to placebo is a key finding, suggesting effective target engagement. This indicates successful sEH inhibition, preserving the upstream epoxide while reducing its conversion to the downstream diol. This increase in the epoxide/diol ratio is consistent with previous findings in monkeys treated with sEH inhibitors (Ulu et al., 2012).

Taken together, several limitations reduce the generalizability of these results. First, given the study design using pigs, the selected inhibitor may have been suboptimal for this species or administered at a dose too low to achieve complete sEH inhibition, despite thorough justification for its use. Second, the LPS-induced lung injury model itself may have been too severe to detect differences in lung injury progression within the studied time frame. Therefore, additional experiments are needed to further evaluate sEH inhibition as a potential treatment for ARDS in porcine models. The structure activity correlations with potency of sEH inhibition in pigs is quite different from rodent and primate species where other sEH inhibitors are far more potent with better pharmacokinetics. We also were limited in needing an sEH inhibitor that could be given by continuous infusion. Future studies should explore additional sEH inhibitors beyond AEPU to develop more effective clinical interventions for ARDS. The current study’s data on IC_50_ and metabolic stability in pig for a range of sEH inhibitors provide a foundation for selecting suitable candidates for such experiments.

## 5 Conclusion

Despite achieving target engagement, administration of the sEH inhibitor AEPU did not attenuate lung injury development in LPS treated pigs under the conditions tested here. The observed increase in the 12 (13)-EpOME/12,13-DiHOME ratio confirmed sEH inhibition, yet AEPU failed to counteract the proinflammatory response observed in the oxylipin profile, nor did it improve clinical outcomes. Possible explanations include species-specific pharmacokinetics and an excessively severe lung injury model. Future studies should investigate alternative sEH inhibitors and refine experimental conditions to further evaluate the therapeutic potential of sEH inhibition in ARDS, as informed by data on candidate drugs presented here.

## Data Availability

The original contributions presented in the study are included in the article/Supplementary Material, further inquiries can be directed to the corresponding author.
